# The miR169b/NFYA1 module from the halophyte *Halostachys caspica* endows salt and drought tolerance in *Arabidopsis* through multi-pathways

**DOI:** 10.3389/fpls.2022.1026421

**Published:** 2023-01-10

**Authors:** Jieyun Ji, Youling Zeng, Suwei Zhang, Fangyuan Chen, Xianfei Hou, Qiang Li

**Affiliations:** ^1^ Xinjiang Key Laboratory of Biological Resources and Genetic Engineering, College of Life Science and Technology, Xinjiang University, Urumqi, sChina; ^2^ Institute of Economic Crops, Xinjiang Academy of Agricultural Sciences, Urumqi, China

**Keywords:** miR169b/NFYA1 module, salt stress, drought stress, *Halostachys caspica*, regulatory mechanism

## Abstract

Salt and drought are the major abiotic stress factors plaguing plant growth, development and crop yields. Certain abiotic-stress tolerant plants have developed special mechanisms for adapting to adverse environments in the long process of evolution. Elucidating the molecular mechanisms by which they can exert resistance to abiotic stresses is beneficial for breeding new cultivars to guide agricultural production. *Halostachys caspica*, a perennial halophyte belonging to *Halostachys* in Amaranthaceae, is extremely tolerant to harsh environments, which is commonly grown in the saline-alkali arid desert area of Northwest, China. However, the molecular mechanism of stress tolerance is unclear. Nuclear Factor Y-A (NFYA) is a transcription factor that regulates the expression of downstream genes in plant response to adverse environments. It has also been reported that some members of the NFYA family are the main targets of miR169 in plants. In this study, we mainly focused on exploring the functions and preliminary mechanism of the miR169b/NFYA1 module from *H. caspica* to abiotic stress. The main results showed that RLM-RACE technology validated that *HcNFYA1* was targeted by HcmiR169b, qRT-PCR revealed that HcmiR169b was repressed and *HcNFYA1* was induced in the *H. caspica* branches under various abiotic stress as well ABA treatment and *Arabidopsis* stable transformation platform with molecular methods was applied to elucidate that the HcmiR169b/HcNFYA1 module conferred the salt and drought tolerance to plants by enhancing ABA synthesis and ABA signal transduction pathways, maintaining ROS homeostasis and the stability of cell membrane. *HcNFYA1* is expected to be a candidate gene to improve plant resistance to salt and drought stresses.

## Introduction

Environmental stresses such as drought and salinity significantly affect plant’ s physiological processes, limit the distribution of plants, and reduce crop production (Bailey-Serres et al., 2019; Zhang et al., 2020). Plants have evolved interrelated regulatory pathways that enable them to respond promptly and adapt to various environmental stresses (Gong et al., 2020). The presence of critical factors in these pathways can enhance plant resistance to different stresses by maintaining the stability of cell membranes and increasing their ability to scavenge reactive oxygen species (ROS), which may be an appropriate target for crop improvement (Miller et al., 2010; Nadarajah, 2020; Zhang et al., 2022).

Due to global climate change, drought has become one of the serious stresses limiting crop yields (Gupta et al., 2020). During soil water deficits, osmotic stress causes plants to express genes, *NCEDs* and *ABAs*, involved in ABA synthesis, which increases the amount of ABA within the tissues (Waadt et al., 2022; Kuromori et al., 2022). When ABA is present, the ABA receptor proteins PYR/PYLs can bind to the protein phosphatase PP2Cs and inhibit their activities, and thereby derepress PP2Cs’ inhibitory effect on the protein kinase SnRK2 (Sah et al., 2016). SnRK2s can phosphorylate transcription factors ABFs and ABI3/5 to activate ABA-responsive genes, which contain ABRE elements in the promoter and their proteins can provide plants with greater tolerance to osmotic stress through different mechanisms, including the closure of stomata (Hsu et al., 2021). Salt stress has also become a major factor reducing crop yields due to land salinization. As plants are exposed to high salt environments, they are poisoned by harmful ions (such as Na^+^) in addition to osmotic stress, and it is widely known certain proteins (SOS, NHX) have been evolved to exclude Na^+^ to the outside of cells and partition sodium ions into vacuoles (Zelm et al., 2020).

MicroRNA (miRNA) is a small non-coding RNA that regulates its complementary target genes by cleaving mRNA or inhibiting translation (Bartel, 2009; Yu et al., 2017). In plants, regulatory modules composed of miRNAs and their target genes play important roles in physiological and biochemical processes (Song et al., 2019; Samad et al., 2017; Pagano et al., 2021). miR169 is one of the largest miRNA families in plants, and its target genes mainly encode the transcription factor nuclear factor Y-A (NFYA) (Luan et al., 2014; Chaves-Sanjuan et al., 2021; Wang et al., 2022). NFYA proteins form trimers with NFYB/C and then bind to CCAAT elements in downstream gene promoters to influence gene expression (Laloum et al., 2013; Zanetti et al., 2017).

The miR169/NFYA module regulates plant growth and development (Luan et al., 2014; Chaves-Sanjuan et al., 2021; Wang et al., 2022). Overexpression of *miR169d* promoted early flowering by targeting *AtNFYA2*; overexpression of *AtNFYA8* was reported to delay flowering in *Arabidopsis thaliana* (Xu et al., 2014; Zhao et al., 2020). However, more studies have focused on the function of miR169/NFYA module in adversity stress (Li et al., 2008). The suppression of *Arabidopsis* miR169a/c expression led to an up-regulation of its target gene, *AtNFYA5*, resulting in drought resistance. *GmNFYA3*, the gene targeted by GmmiR169c in soybean (*Glycine max*), confers drought tolerance, while GmmiR169c reduced drought resistance in *Arabidopsis* by targeting the cleavage of *AtNFYA1/5* (Ni et al., 2013; Yu et al., 2019). Salt stress suppressed the expression of maize (*Zea mays*) ZmmiR169q, and up-regulated its target gene *ZmNFYA8*, finally enhancing salt tolerance in maize by attenuating ROS-induced toxicity (Xing et al., 2021). In contrast, tomato (*Solanum lycopersicum*) SlymiR169c and poplar (*Populus trichocarpa*) PtmiR169o enhanced the plant’s drought resistance (Zhang et al., 2011; Jiao et al., 2021). The miR169/NFYA module in plants is not functionally conserved in response to abiotic stresses.

*Halostachys caspica* is a perennial shrub in Amaranthaceae, which can grow in the extremely arid and saline-alkali environments. In our previous study, the expression of miR169b was significantly different in the small RNA libraries of the *Halostachys caspica* roots under high salinity and *NFYA1* was the potential target of this miRNA using this species’ transcriptome data by bioinformatic prediction. (Yang et al., 2015). However, the miR169b/NFYA1 module has not been studied, and its role has not still been elucidated in *H. caspica*. In this work, we first experimentally validated that *HcNFYA1* is the real target of HcmiR169b, and explored their expression patterns in *H. caspica* under various abiotic-stresses. The functions and regulatory mechanism of the HcmiR169b and HcNFYA1 module were investigated by generating *HcmiR169b*/*HcNFYA1* heterologously expressed *Arabidopsis thaliana*.

## Materials and methods

### *H. caspica* culture and stress treatments

*H. caspica* seeds were harvested from extremely saline-alkali and arid areas in the Gurbantunggut Desert in Xinjiang, China. Healthy seeds were sown in pots with the substrate (perlite: vermiculite: flower soil = 1:1:3) and cultivated under natural light and suitable temperature (25°C - 28°C). Eight-week-old *H. caspica* plants were exposed to 600 mM NaCl, 1000 mM mannitol, 0 °C freezing stress, 100 μM methyl viologen (MV) and 300 μM abscisic acid (ABA) for 0, 3 and 24 h, and assimilating branches were taken and put in liquid nitrogen for subsequent qRT-PCR assays.

### Cloning and bioinformatics analysis of *HcmiR169b* precursor and *HcNFYA1*

The *HcmiR169b* mature sequence was obtained from a small RNA library derived from *H. caspica* roots (Yang et al., 2015). The *HcmiR169b* precursor sequence was obtained through homologous cloning and 5’-RACE nested amplification using the *H. caspica* cDNA as template. Based on the EST sequence of *HcNFYA1* in the *H. caspica* transcriptome data, the full length of *HcNFYA1* gene was cloned from the *H.caspica* cDNA using the SMARTer RACE 5’/3’ Kit (Takara, Japan).

Amino acid sequences of NFYA family members were downloaded from the PlantTFDB plant transcription factor database (http://planttfdb.gao-lab.org/index.php) (Jin et al., 2017). Multiple comparisons of amino acid sequences were performed using DNAMAN software (LynnonBiosoft, USA). Phylogenetic analysis was applied using the proximity method (1000 replicates) with MEGA 11 software (Mega Limited, New Zealand).

### Validation of HcmiR169b cleavage site in *HcNFYA1* by RLM-RACE

As shown in the **Supplementary Figure S1**, the method in this study was modified according to the RNA ligase mediated (RLM)-cDNA end rapid amplification (RACE) technique developed by (Wang and Fang, 2015). T4 RNA ligase (Ambion, Canada) was used to add an adapter to the 3’ hydroxyl end of *H. caspica* RNA. A specific primer (GSP) complementary to the adapter was used for reverse transcription to obtain cDNA with the adapter sequence. Two rounds of nested PCR amplification was carried based on the cDNA and the second round of amplification products were cloned into the pMD-19T vector for sequencing, and the sequencing results were analyzed to determine the cleavage site of *HcNFYA1* by HcmiR169b. The primers used in this study were listed in **Supplementary Table S1**.

### Detection of gene expression by qRT-PCR

Total RNAs were extracted from plant samples by RNA prep pure Plant Kit (Tiangen, China). The stem-loop method was used for microRNA reverse transcription, with *HcU6* and *AtU6* serving as internal controls (Chen et al., 2005), and protein-coding genes were reverse-transcribed using One-Step gDNA Removal and cDNA Synthesis SuperMix (Transgen, China), with *HcUBQ10* and *Atactin* as internal controls (Zhang et al., 2015). qRT-PCR was performed in a CFX96 Touch Real-Time PCR System (Bio-Rad, USA) using a PerfectStart Green qPCR SuperMix (Transgen, China) with three biological replicates per sample. The relative expression of genes was calculated by 2^−ΔΔCT^ comparison method (Livak and Schmittgen, 2001). The primers were shown in **Supplementary Table S1**.

### Subcellular localization and transcriptional activation analysis of HcNFYA1

The *HcNFYA1* open reading frame (ORF) region was inserted into the plant expression vector pCAMBIA1301-1-GFP (Liu et al., 2009). HcNFYA1 was fused to GFP for expression and transformed into onion epidermal cells using the *Agrobacterium* GV3101-mediated transient transformation system. Onion epidermal cells were stained with DAPI and observed under a LSM800 Confocal Microscope (Zeiss, Germany).

The ORF region of *HcNFYA1* was constructed into the pGBKT7 vector containing the GAL4 DNA binding domain. The recombinant plasmid was transformed into the Y2H Gold yeast strain. Transformants were screened in SD/-Trp-His medium containing X-α-Gal and HcTOE3 was used as a positive control (Yin et al., 2021). The primers were listed in **Supplementary Table S1**.

### Generation of transgenic *Arabidopsis* and stress treatment

The precursor sequence of *HcmiR169b* and the ORF region of *HcNFYA1* were constructed into plant expression vector pCAMBIA2300, and transformed into *Arabidopsis* wild type (Columbia) by inflorescence infection (Zhang et al., 2006). Positive lines were screened by kanamycin primarily. The selected T_3_ generation transgene *Arabidopsis* lines with single copy were verified by genomic PCR and qRT-PCR.

*Arabidopsis* seeds were sterilized with sodium hypochlorite (10%) and 75% ethanol (90%) for 5 min and sown in 1/2 MS medium. After 3 days of vernalization at 4°C with a 16 h/8 h light/dark photoperiod, petri dishes with plants were put in the growing chamber at 22°C. After two weeks of growth, the plants were transferred to pots filled with the substrate (perlite: vermiculite: flower soil = 1:1:3) for cultivation.

*Arabidopsis* germination experiments were conducted in 1/2 MS medium containing NaCl (125 mM), mannitol (250 mM), and ABA (0.5 μM, 0.75 μM). The seed germination rates were recorded daily, and the cotyledon greening rates were measured after 7 days.

For the salt treatment, four-week-old *Arabidopsis* plants were irrigated with 300 mM NaCl for 7 days, photographed, dried at 80°C for 24 hours, and weighed for the dry weight (10 biological replicates). 300 mM NaCl-treated *Arabidopsis* leaves were collected for qRT-PCR (24 h) and measuring some physiological and biochemical parameters (3 d).

For the drought treatment, *Arabidopsis* plants were planted in pots containing the same substrate weight and re-watered for 3 days after stopping irrigation for 7 or 9 days. The plants were photographed and counted for survival numbers (three independent experiments with 40 biological replicates each). *Arabidopsis* leaves were collected when irrigation was stopped for 5 days for physiological index testing. Leaves were taken for qRT-PCR analysis after 24 h of treatment when *Arabidopsis* was irrigated with 20% PEG6000.

### Determination of physiological and biochemical indicators

Adult *Arabidopsis* leaves were submerged with 1 mg/mL of Evans blue, DAB, and NBT solution and stained for 2 h at 37°C in the dark. The leaves were placed in absolute alcohol at boiling temperature for 30 min to remove chlorophyll. 10 blades were run per treatment.

Whole *Arabidopsis* plants were soaked in 50 ml ultrapure water for 24 h. The solution conductivity (C1) was measured and the solution conductivity (C2) was measured again after boiling for 1 h using a Conductivity Meter (DSS-307, China). C1/C2×100% was calculated as the relative electrolyte leakage.

The measurements of chlorophyll, MDA, H_2_O_2_, ${\text{O}}_{2}^{-}$ and proline contents as well as the detection of POD, APX and SOD enzyme activities were performed according to the manufacturer’s instructions (Solarbio, China). Soluble protein content was determined using the BCA Soluble Protein Content Kit (Addison, China). The content of ABA was determined by ABA ELISA assay kit (Saipei, China). Per sample had three biological replicates for each treatment, and 0.1 g of *Arabidopsis* leaves were collected for each biological replicate.

### Analysis of water loss and stomatal aperture of *Arabidopsis* leaves

*Arabidopsis* leaves were taken and placed in an area with good airflow. The initial fresh weight (W_0_) and the fresh weight of the leaves were measured every 5-30 minutes (W_t_), and 100%-W_t_/W_0_ was called as the water loss rate.

*Arabidopsis* leaves were immersed in stomatal opening buffer (5 mM MES, 10 mM KCl, 50 mM CaCl_2_, pH 5.6) for 2 h, transferred to a solution containing 0 mM/300 mM mannitol or 0 μM/30 μM ABA for 2 h and then observed under a microscope for photographs. The aspect ratio was analyzed using Image J software (National Institutes of Health, USA). Three *Arabidopsis* leaves were run for each treatment, and at least 50 clear stomata in the field of view were obtained for each leaf.

### Statistical analysis

SPSS Statistics 20 (IBM, USA) was employed for data analysis. All experiments were performed at least 3 times. Experimental data of gene expression in *H. caspica* and aspect ratio with *Arabidopsis* leaves for stomatal aperture analysis were assessed by Student’s t-test (*p<0.05, **p<0.01, ***p<0.001, ****p<0.0001). All other data were assessed using Duncan’s test (*p*<0.05).

## Results

### Expression patterns of miR169b and its targeted gene *NFYA1* in *Halostachys caspica* under abiotic stresses

**Figure 1:**
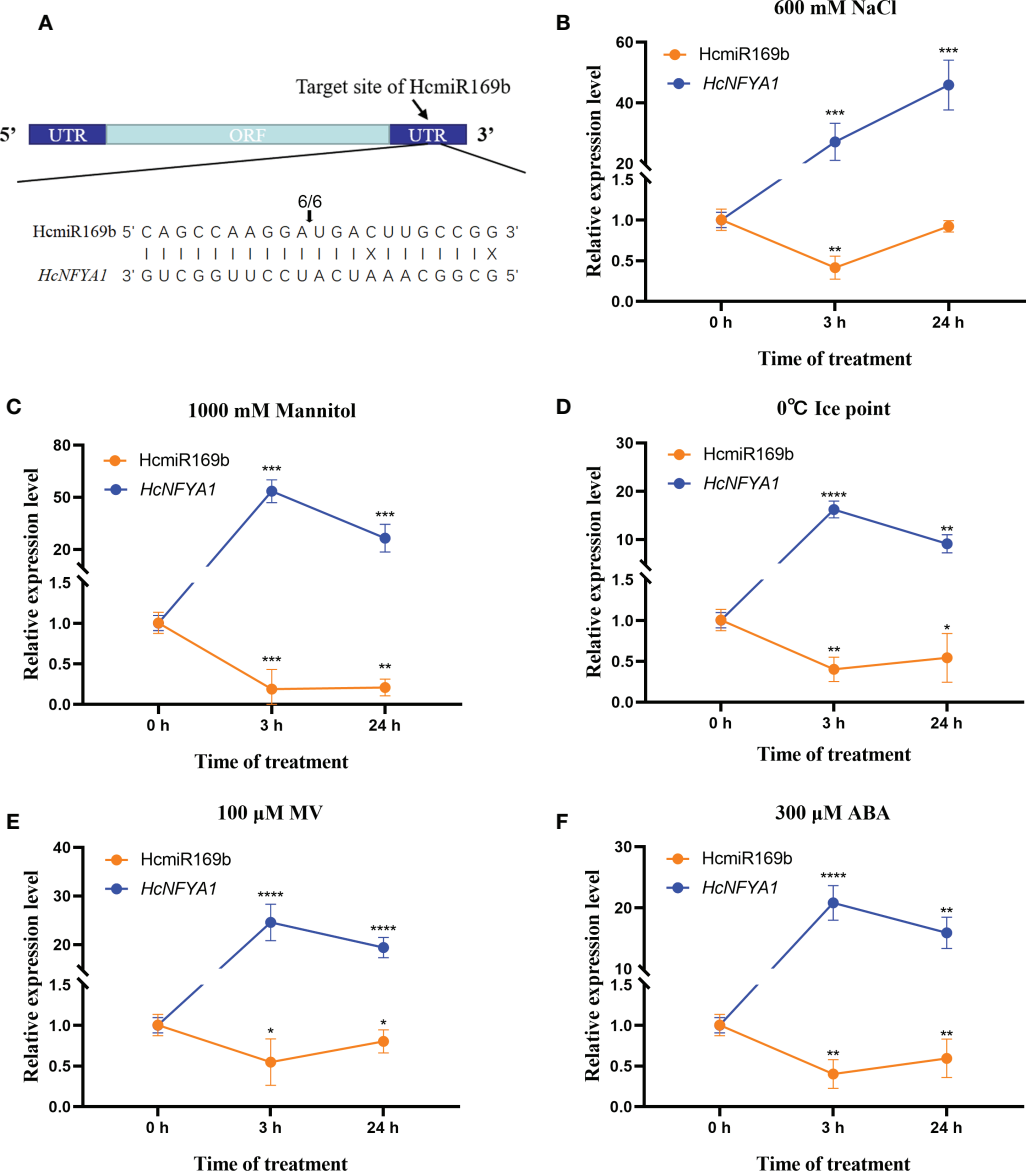
Targeting relationship between *Halostachys caspica* miR169b and *NFYA1* and the expression patterns of both genes under abiotic stress. **(A)** RLM-RACE identified the targeted cleavage of *HcNFYA1* by HcmiR169b. Arrows indicated specific cleavage site, and the number on the arrow indicated the number of independent clones for detecting the cleavage site. **(B-F)** The expression of mature miR169b and *NFYA1* in assimilating branches of *Halostachys caspica* under salt **(B)**, simulated drought **(C)**, cold **(D)**, oxidative stress **(E)** and exogenous hormone ABA **(F)**. *HcU6* and *HcUBQ10* were used as internal controls. All data were represented as the means ± SD of three biological replicates. Asterisks indicated significantly different transcriptional levels compared to 0 h (Student’s t-test, *p<0.05, **p<0.01, ***p<0.001, ****p<0.0001).

*HcNFYA1* was predicted to be targeted by HcmiR169b in our previous work (Yang et al., 2015). Here, RLM-RACE experiment was conducted to verify their targeting between HcmiR169b and *HcNFYA1* (Rhoades et al., 2002; Wu et al., 2012). The results of agarose gel electrophoresis were shown in the **Supplementary Figure S2**. The final products by sequencing indicated that HcmiR169b cleaves the 3’UTR region of *HcNFYA1* with 100% (6/6) efficiency (**Figure 1A**).

Eight-week-old *H. caspica* were exposed to salt (**Figure 1B**), drought (**Figure 1C**), cold (**Figure 1D**), and oxidative stress (**Figure 1E**) as well as exogenous hormone ABA (**Figure 1F**). After 3 and 24 hours of treatment, individual assimilating branches were extracted and used for qRT-PCR analysis. The results showed that the expressions of HcmiR169b and *HcNFYA1* presented a significant negative correlation, HcmiR169b was significantly inhibited and *HcNFYA1* was notably induced under different abiotic stress treatments. HcmiR169b and *HcNFYA1* appeared to respond to various abiotic stresses, including salt and drought stress; *HcNFYA1* might be controlled by HcmiR169b under these adverse conditions.

### HcNFYA1 acts as a transcription factor

The coding sequence of *HcNFYA1* is 903 bp in length, and its encoded protein contains 300 amino acids. HcNFYA1 protein contained the binding sites for NFYB/C and CCAAT (**Supplementary Figure S3A**). Based on a phylogenetic analysis of conserved amino acids found in HcNFYA1 and members of the NFYA family of other plant species, including *Arabidopsis*, soybean, maize, rice, and *Beta vulgaris*, HcNFYA1 was clustered with BvNFYA1 (**Supplementary Figure S3B**).

**Figure 2:**
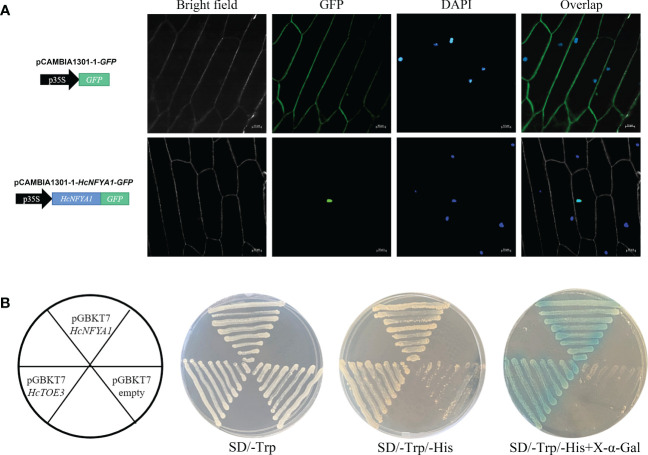
Characteristic analysis of transcription factor HcNFYA1. **(A)** Localization of HcNFYA1-GFP fusion protein in onion epidermal cells, DAPI was used as a nuclear marker. **(B)** Transcriptional activation analysis of HcNFYA1 in yeast expression system. HcTOE3 was used as a positive control.

To determine the subcellular localization of HcNFYA1, the HcNFYA1-GFP fusion protein was expressed in the onion epidermis using an *Agrobacterium*-mediated transient transformation system. Under laser confocal microscopy, the fluorescent signal of HcNFYA1-GFP overlapped with the 4-diamino-2-phenylindole (DAPI) signal were observed and appeared only in the nucleus (**Figure 2A**). This suggested that *HcNFYA1* was translated and then translocated into the nucleus to function.

To identify whether HcNFYA1 functions as a transcription factor. The ORF region of *HcNFYA1* was constructed into the yeast BD expression vector pGBKT7 and transformed into Y2H Gold cells. The HcNFYA1 protein, like the positive control (Yin et al., 2021), activated the expression of a downstream reporter gene, the protein encoded by this gene caused X-α-Gal to degrade and made transformed yeast show blue color (**Figure 2B**), indicating that HcNFYA1 had transcriptional activation activity.

### *Halostachys caspica* miR169b/NFYA1 module affects the germination of *Arabidopsis* seeds under salt and drought stress

**Figure 3:**
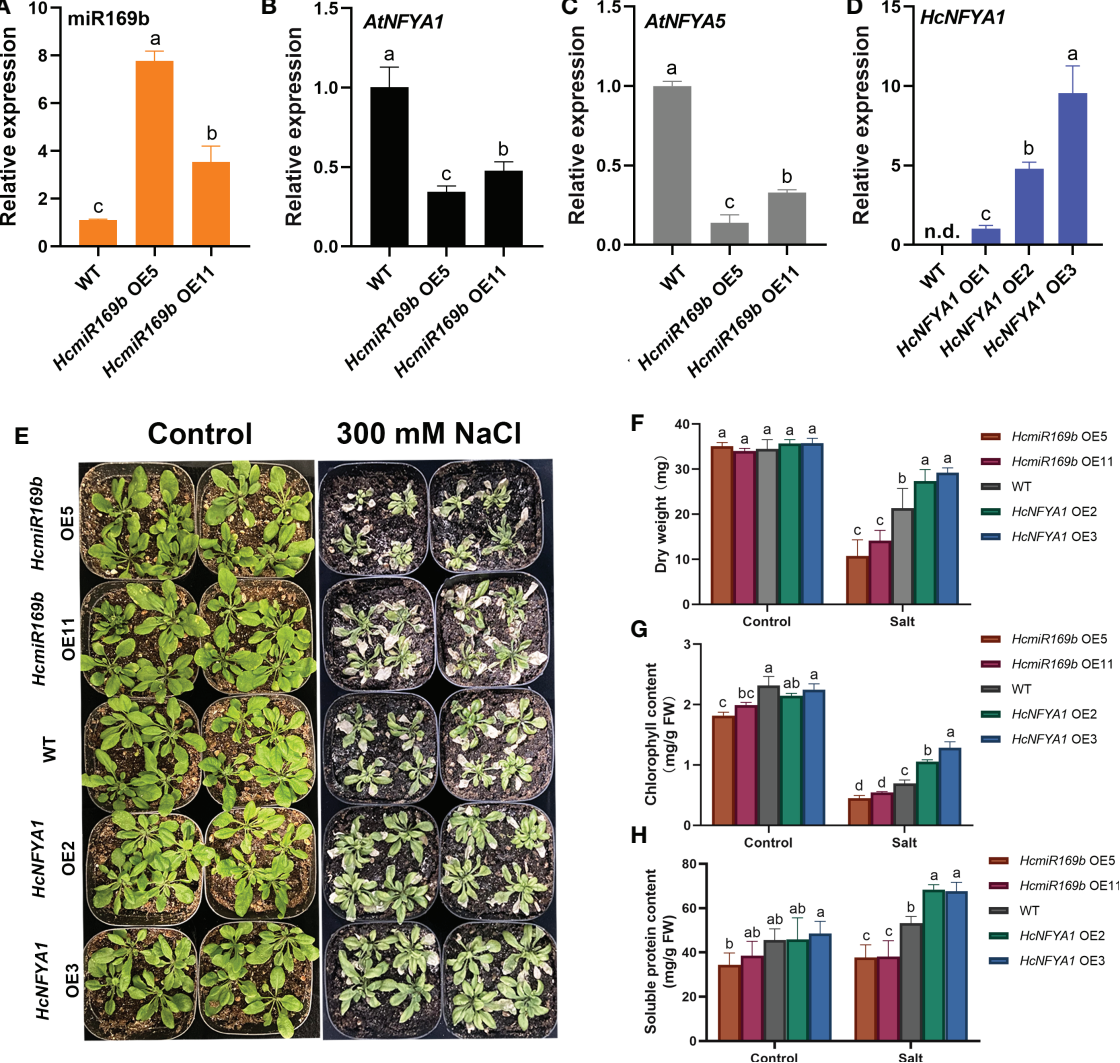
Functional analysis of *Halostachys caspica* miR169b/NFYA1 module under salt stress. **(A-C)** Relative expression of miR169b **(A)**, *AtNFYA1* **(B)** and *AtNFYA5* **(C)** in wild-type and *HcmiR169b* heterologous expressed *Arabidopsis*, the expression level of wild-type was adjusted to 1. **(D)** Relative expression of *HcNFYA1* in wild-type and *HcNFYA1* heterologous expressed *Arabidopsis*, adjusting the expression level of *HcNFYA1* OE1 to 1. **(E)** Phenotype of adult *Arabidopsis* under 300 mM NaCl stress for 7 d. **(F-H)** The aboveground dry weight **(F)**, chlorophyll content **(G)** and soluble protein content **(H)** of *Arabidopsis* under salt stress. Values for aboveground dry weight represented the means ± SD of 10 biological replicates, and values for other data represented the means ± SD of three biological replicates. Different letters indicated significant differences in the detected values of various types of *Arabidopsis* between treatments (Duncan’s multiple range test, *p*<0.05).

To elucidate the function of the HcmiR169b/HcNFYA1 module in abiotic stress, we generated 35S:*HcmiR169b* and 35S:*HcNFYA1* transgenic *Arabidopsis* homozygous lines with single copy by the methods of inflorescence infection and kanamycine screening. Based on the results of qRT-PCR assay for T_3_ generation of transgenic plants, two individual transgenic lines (*HcmiR169b* OE5 and *HcmiR169b* OE11, *HcNFYA1* OE2 and *HcNFYA1* OE3) were selected for subsequent experiments; both of transgenic lines had high expressions for respectively transformed genes (**Figure 3A, D**). Because of the highly conserved mature sequences between HcmiR169b and AtmiR169b, a portion of AtmiR169b expression was also detected possibly (**Supplementary Figure S4**). The expressions of its predicted target gene *AtNFYA1/5* were reduced in 35S:*HcmiR169b Arabidopsis* by qRT-PCR assay (**Figure 3B, C**).

The seed germination was consistent between 35S:*HcmiR169b Arabidopsis* and WT on 1/2 MS medium (**Supplementary Figure S5A**). However, the germination rate of 35S:*HcmiR169b* seeds was lower when NaCl (125 mM) or mannitol (250 mM) was added to the medium in comparison with the WT (**Supplementary Figure S5B, C**), suggesting that HcmiR169b made *Arabidopsis* more sensitive to salt and drought stresses during the stage of seed germination. Compared with the WT, 35S*:HcNFYA1 Arabidopsis* seeds in 1/2 MS medium were exhibited slightly delayed germination (**Supplementary Figure S5D**). When NaCl or mannitol was added to the medium, the germination rate of 35S:*HcNFYA1 Arabidopsis* seeds was decreased compared to the WT (**Supplementary Figure S5E, F**). Overexpression of *HcNFYA1* inhibited *Arabidopsis* seed germination under salt- and drought- stressed conditions.

### *Halostachys caspica* miR169b/NFYA1 module regulates salt tolerance in *Arabidopsis*

Under salt stress of 300 mM NaCl, 35S:*HcmiR169b Arabidopsis* grew smaller and the leaves wilted more severely than WT, whereas 35S:*HcNFYA1 Arabidopsis* grew larger, leaves wilted less (**Figure 3E**). In addition, under salt stress, the dry weight of aboveground parts (**Figure 3F**), chlorophyll (**Figure 3G**) and soluble protein (**Figure 3H**) contents were lower in 35S:*HcmiR169b Arabidopsis* and higher in 35S:*HcNFYA1 Arabidopsis*, compared to those in the WT. In conclusion, HcmiR169b negatively regulated salt tolerance in *Arabidopsis*, whereas HcNFYA1 was positive in regulating salt tolerance in *Arabidopsis*.

### *Halostachys caspica* miR169b/NFYA1 module regulates drought tolerance in *Arabidopsis*

**Figure 4:**
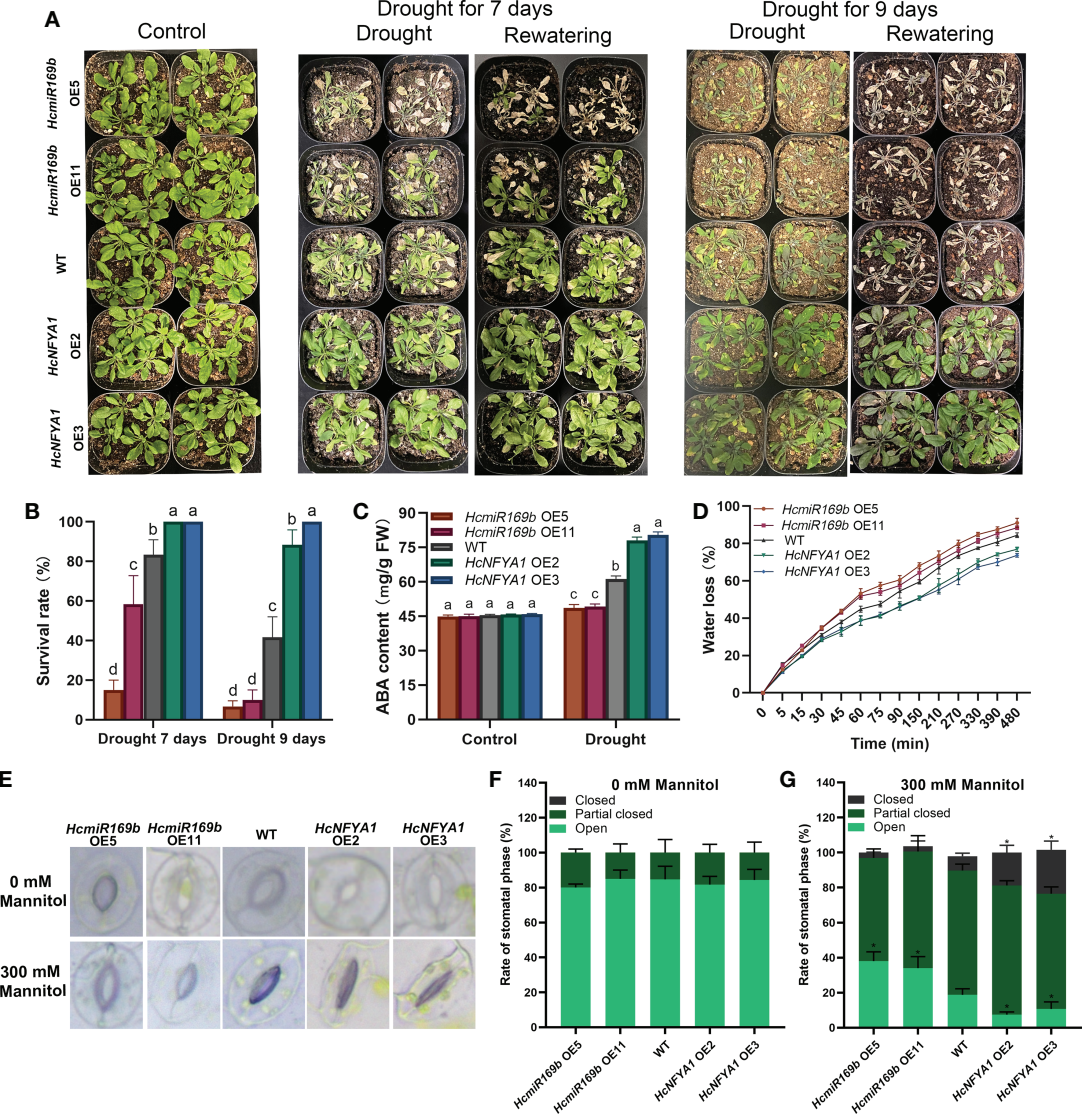
Functional analysis of *Halostachys caspica* miR169b/NFYA1 module under drought stress. **(A, B)** Phenotype **(A)** and survival **(B)** of adult *Arabidopsis* after 7 or 9 days of natual drought and 3 days of rewatering. **(C)** ABA content in *Arabidopsis* under control and drought stress. **(D)** Determination of water loss in detached leaves of *Arabidopsis*. **(E-G)** Stomatal apertures of *Arabidopsis* leaves under 0 and 300 mM mannitol (simulated drought) treatment were photographed **(E)** and the percentage of various stomata **(F, G)**. Data were shown as the means ± SD of three independent experiments. Different letters indicated significant differences in survival rate and ABA content of each type of *Arabidopsis* between treatments (Duncan’s multiple range test, *p*<0.05), asterisk indicated a significant difference in the percentage of this stomatal type compared to WT (Student’s t-test, *p<0.05).

To examine the role of *Halostachys caspica* miR169b/NFYA1 in response to drought stress, four-week-old *Arabidopsis* were stopped from irrigation untill different phenotypes emerged among them. Almost 7 days after ceasing to irrigate, 35S:*HcmiR169b Arabidopsis* showed leaf drying, and its survival rate after 3 days of rewatering was significantly lower than that of WT. Continuing with no irrigation for 9 days, 35S:*HcmiR169b Arabidopsis* leaves had completely dried out and wild-type *Arabidopsis* leaves were wilted, whereas 35S:*HcNFYA1 Arabidopsis* was still growing well. At this point, 3 days after rewatering, the survival rate of 35S:*HcmiR169b Arabidopsis* was already below 20%, 50% for wild-type *Arabidopsis*, and up to 80% for 35S:*HcNFYA1 Arabidopsis* (**Figure 4A, B**). Under drought stress, the drought-responding positive regulatory hormone ABA content was the highest in 35S:*HcNFYA1 Arabidopsis* and the lowest in 35S:*HcmiR169b* (**Figure 4C**). HcmiR169b appeared to be a negative regulator of drought resistance in *Arabidopsis*, whereas HcNFYA1 was a positive regulator.

Water loss was faster in detached leaves of 35S:*HcmiR169b Arabidopsis* and slower in leaves of 35S:*HcNFYA1 Arabidopsis* compared with WT (**Figure 4D**). In detached leaves of *Arabidopsis*, the rate of water loss was primarily dependent on the degree of stomatal opening. After treatment with 300 mM mannitol, 35S:*HcmiR169b Arabidopsis* had significantly more open stomata than WT, and 35S:*HcNFYA1 Arabidopsis* had more completely closed stomata than WT (**Figure 4E-G**). This suggested that the HcmiR169b/HcNFYA1 module may regulate drought tolerance in *Arabidopsi*s through stomatal activity.

### *Halostachys caspica* miR169b/NFYA1 module regulates the stability of plant cell membrane systems and resistance to oxidative stress

**Figure 5:**
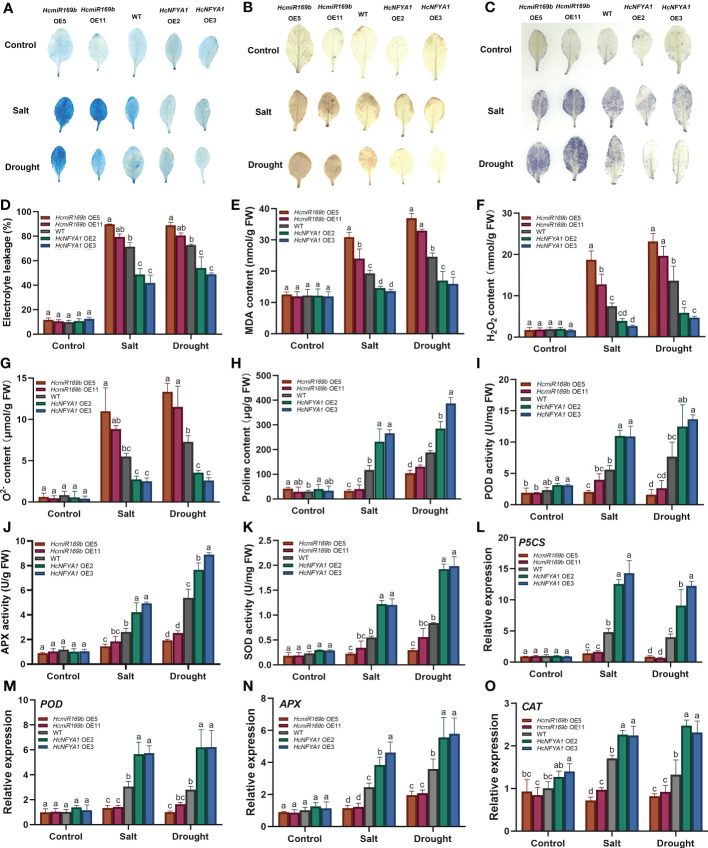
The performance of WT and two transgenic (*HcmiR169b*, *HcNFYA1* OE) *Arabidopsis* lines under salt and drought stress. **(A-C)** Evans blue **(A)**, DAB **(B)** and NBT **(C)** staining of adult *Arabidopsis* leaves under control, salt (300 mM NaCl) and drought (stop the irrigation) stress. **(D)** Electrolyte leakage assay. **(E-H)** MDA**(E)**, H_2_O_2_ **(F)**, ${\text{O}}_{2}^{-}$ **(G)**, proline (**H**) contents. (**I–K**) POD (**I**), APX **(J)**, SOD **(K)** enzyme activities. **(L-O)** Relative expression of *P5CS* **(L)**, *POD* **(M)**, *APX* **(N)**, *CAT* **(O)** genes. All data represented the means ± SD of three biological replicates. Different letters indicated significant differences in the detected values of various types of *Arabidopsis* between treatments (Duncan’s multiple range test, *p*<0.05).

Adversity stress leads to disruption of plant cell membrane and ROS homeostasis. We monitored the integrity of cell membranes in various types of *Arabidopsis* under salt and drought stress based on the Evans blue staining (**Figure 5A**), electrolyte leakage (**Figure 5D**) and malondialdehyde (MDA) content (**Figure 5E**) assays. The results showed that 35S:*HcmiR169b Arabidopsis* cell membrane was more severely damaged compared to the WT, while 35S:*HcNFYA1 Arabidopsis* cell membrane integrity was better. Diaminobenzidine (DAB) and nitro-blue tetrazolium (NBT) staining (**Figure 5B, C**), H_2_O_2_ and ${\text{O}}_{2}^{-}$ content (**Figure 5F, G**) measurements with leaves in various *Arabidopsis* were performed. The results showed that 35S:*HcmiR169b Arabidopsis* accumulated more ROS and 35S:*HcNFYA1 Arabidopsis* increased less ROS compared to the WT under salt and drought stress.

Proline (Pro)is used as an osmoregulatory substance to keep the osmotic pressure stability in plants and maintain the integrity of the cell membrane (Székely et al., 2008). The Pro content (**Figure 5H**) and the expression of its key synthetic enzyme gene *P5CS* (**Figure 5L**) were lower in 35S:*HcmiR169b Arabidopsis* than those in the WT under salt and drought stress, and the opposite results were obtained from 35S:*HcNFYA1 Arabidopsis*. Antioxidant enzymes play an important role in scavenging plant ROS species, so the activities of antioxidant enzymes (**Figure 5I-K**) and their transcription levels of relative synthetic enzyme genes (**Figure 5M-O**) were examined in *Arabidopsis*. Under salt and drought stress, 35S:*HcmiR169b Arabidopsis* had the lowest antioxidant enzyme activities and gene expression at both protein and RNA levels, whereas 35S:*HcNFYA1 Arabidopsis* had the highest antioxidant enzyme activities, and WT *Arabidopsis* was in the middle. Under the control conditions, there was no significant difference in physiological indices and gene transcription levels between the transgenic (*HcmiR169b*, *HcNFYA1* OE) *Arabidopsis* and the WT.

### Expression analysis of stress-responsive genes in *Halostachys caspica* miR169b/NFYA1 module

**Figure 6:**
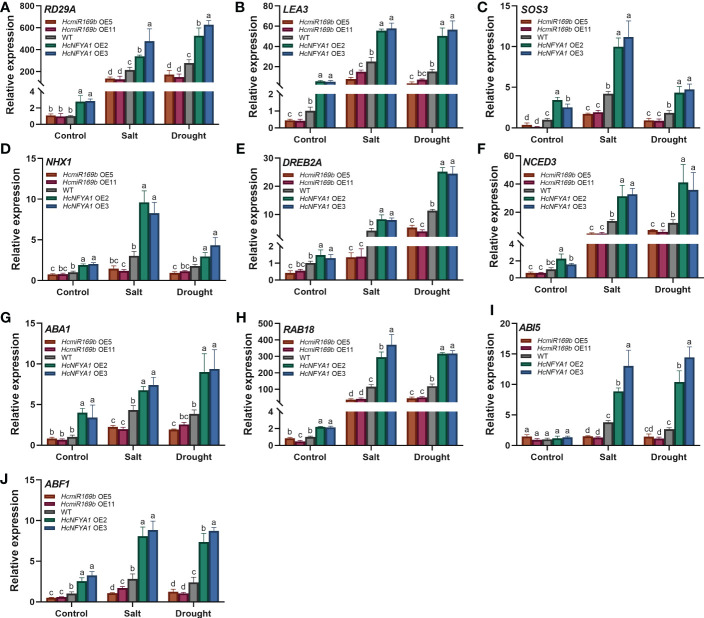
Expression of downstream stress-responsive genes in WT and two transgenic (*HcmiR169b*, *HcNFYA1* OE) *Arabidopsis* under salt and drought stress. **(A-J)** The relative expression levels of *RD29A* **(A)**, *LEA3* **(B)**, *SOS3* **(C)**, *NHX1* **(D)**, *DREB2A* **(E)**, *NCED3* **(F)**, *ABA1* **(G)**, *RAB18* **(H)**, *ABI5* **(I)** and *ABF1* **(J)** in adult *Arabidopsis* under the control, salt (300 mM NaCl) and drought (20% PEG6000) stress. All data represented the means ± SD of three biological replicates. Different letters indicated significant differences in the detected values of various types of *Arabidopsis* between treatments (Duncan’s multiple range test, *p*<0.05).

To investigate the regulatory mechanism in which the *Halostachys caspica* miR169b/NFYA1 module functions under salt and drought stress, we selected classical stress-responsive genes (*RD29A*, *LEA3*), salt stress-related genes (*SOS3*, *NHX1*), drought stress-related gene (*DREB2A*), ABA synthesis genes (*NCED3*, *ABA1*) and ABA signaling pathway positively regulatory genes (*ABF1*, *RAB18*, *ABI5*) for further analysis. Notably, in the controls, three genes, *LEA3*, *SOS3*, and *ABF1*, were significantly less expressed in 35S:*HcmiR169b Arabidopsis* and much more expressed in 35S:*HcNFYA1 Arabidopsis* than those in the WT, and all of them have CCAAT elements in their promoters (**Figure 6B, C, J**). Among them, *ABF1* was directly regulated by NFYA family members in soybean (Yu et al., 2021).

Under salt and drought stress conditions, all genes were significantly more expressed in 35S:*HcNFYA1 Arabidopsis* than those in the WT, and the opposite was observed in the 35S:*HcmiR169b Arabidopsis* (**Figure 6**). According to these results, HcNFYA1 can confer salt and drought tolerance to plants by activating downstream responsive genes; HcmiR169b reduced salt and drought resistance by silencing these pathways these genes involved in.

### *Halostachys caspica* miR169b/NFYA1 module regulates plant response to exogenous hormone ABA

**Figure 7:**
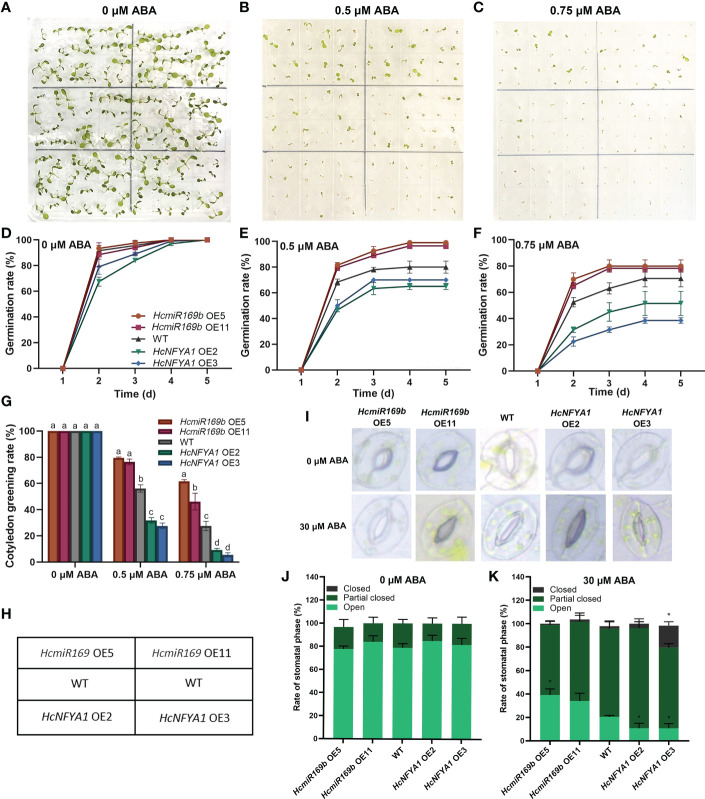
*Halostachys caspica* miR169b/NFYA1 module responds to the exogenous hormone ABA. **(A-G)** Seed germination phenotypes **(A–C)**, germination curves **(D-F)**, and cotyledon greening rate **(G)** of *Arabidopsis* under 0 μM, 0.5 μM, and 0.75 μM ABA treatments. **(H)** Layout designs of figure A-C. **(I-K)** Stomatal aperture photographs **(I)** and percentage of various stomata **(J, K)** of *Arabidopsis* leaves under 0 μM ABA and 30 μM ABA treatments. Data were shown as the means ± SD of three biological replicates. Different letters indicated significant differences in cotyledon greening rate of each type of *Arabidopsis* between treatments (Duncan’s multiple range test, *p*<0.05), asterisk indicated a significant difference in the percentage of this stomatal type compared with WT (Student’s t-test, *p<0.05).

Exogenous hormone ABA reduces the seed germination rate of plants (Sah et al., 2016). To explore how the miR169b/NFYA1 module is involved in ABA signaling, the seeds of various types of *Arabidopsis* lines were sown on 1/2 MS medium containing 0, 0.5, and 0.75 μM ABA to observe their germination and cotyledon greening rates. Several types of *Arabidopsis* were capable of reaching 100% germination and cotyledon greening on ABA-free media; however, the germination rate and cotyledon greening rate of 35S:*HcmiR169b Arabidopsis* were significantly higher with exogenous hormone ABA treatments than without ABA treatment, while these growth parameters of 35S:*HcNFYA1 Arabidopsis* were significantly lower than WT (**Figure 7A-H**). ABA also induces the closure of plant stomata. Under 30 μM ABA treatment, stomatal closure in the leaves of 35S:*HcmiR169b Arabidopsis* was lower and stomatal closure of 35S:*HcNFYA1 Arabidopsis* was higher compared with WT (**Figure 7I-K**). Accordingly, HcmiR169b inhibited the ABA signaling pathway in *Arabidopsis*, while HcNFYA1 activated this pathway.

## Discussion

**Figure 8:**
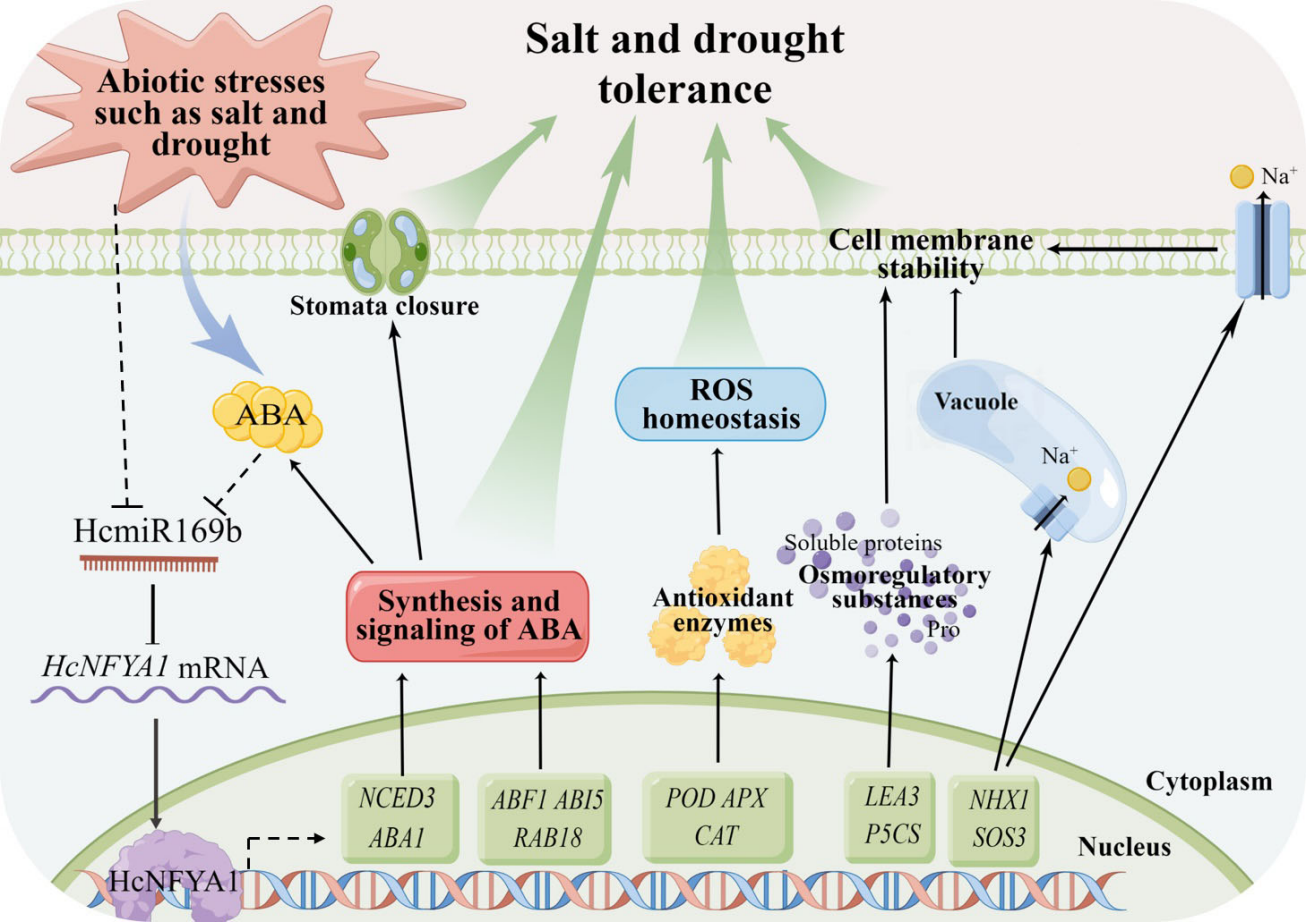
*Halostachys caspica* miR169b/NFYA1 module participates in the regulation network of plant tolerance to salt and drought stresses.

*Halostachys caspica* grown in the saline-alkali arid land for a long history, has developed extreme resistance to harsh environments. We have reported that miRNAs play an essential role in stress tolerance in this species (Yang et al., 2015). Using the *Arabidopsis* stable transformation platform and molecular methods, we revealed such a resistance mechanism on abiotic stress—*Halostachys caspica* miR169b-targeted NFYA1 improves plant tolerance to salt and drought stresses through enhancing ABA synthesis and the associated signaling pathways, maintaining ROS homeostasis and the cell membrane integrity (**Figure 8**).

The miR169 family is the largest and most conserved miRNA family in plants. *NFYA*, targeted by miR169, has been validated in maize, soybean, and oilseed rape. In these species, miR169 expression are repressed by various stresses such as salt and drought; whereas the opposite pattern is observed for *NFYA* genes (Luan et al., 2014; Yu et al., 2019; Wang et al., 2022). Our present study validated that *HcNFYA1* is a true target of miR169b in *H. caspica* by RLM-RCE technology (**Figure 1A**). Under salt, drought stress and ABA treatment, HcmiR169b expression was inhibited, and its targeted gene *HcNFYA1* was significantly induced in *H. caspica* (**Figure 1B-F**). Thus, miR169/NFYA1 module may play an important role in plant’s adaptation to adversity stresses.

It was reported that the AtmiR169a and GmmiR169c made *Arabidopsis* sensitive to drought stress in adulthood by negative regulating the drought-resistant regulator *AtNFYA5* (Li et al., 2008; Yu et al., 2019). Moreover, these two microRNA mature sequences differ from HcmiR169b by only two bases at the 3’ end. In our study, overexpression of HcmiR169b in *Arabidopsis* also reduced the expression of *AtNFYA5* and *AtNFYA1*, as shown in **Figure 3B, C**. Based on these results, we demonstrated that HcmiR169b conferred *Arabidopsis* sensitivity to salt and drought stress at both the germination and adult stages (**Supplementary Figure S5A-C**, **Figure 3**, **4**).

As shown in the **Supplementary Table S2**, nine NFYA family members have been reported to contribute to plant resistance to salt and drought stress in *Arabidopsis*, soybean, poplar, maize, and rice. (Lee et al., 2015; Lian et al., 2018; Ma et al., 2020a; Ma et al., 2020b). However, the reported members are evolutionarily distant from HcNFYA1 except for AtNFYA1 (**Supplementary Figure S3**). AtNFYA1 inhibits the seed germination of *Arabidopsis* under salt stress by enhancing ABA signaling (Li et al., 2013); this ABA-mediated arrest of seed germination provides plants with an adaptive mechanism to improve survivals under stress conditions (Zhu, 2016). HcNFYA1 also enhanced ABA signaling (**Figure 7**) and stalled the seed germination of *Arabidopsis* under salt and drought stress (**Supplementary Figure S5D-F**). Nevertheless, once the plants reached adulthood, 35S:*HcNFYA1 Arabidopsis* exhibited greater tolerance to salt and drought than the WT and 35S:*HcmiR169b Arabidopsis* (**Figure 3**, **4**).

In recent years, the miR169/NFYA module has received more attention, and its functions have been reported to respond to drought stress in oilseed rape and poplar, and to salinity stress in maize (Li et al., 2021; Jiao et al., 2021; Xing et al., 2021). In this study, we utilized the *Arabidopsis* stable transformation platform and molecular methods to elucidate the action mechanism of the *Halostachys caspica* miR169b/NFYA1 module to confer plant salt tolerance and drought resistance by multiple pathways, including (i) Synthesis and signaling transduction of ABA. Dry conditions and high salinity can cause plants to experience osmotic stress, and ABA is the most significant hormone accumulated in plant response to osmotic stress. To respond to osmotic stress, ABA induced leaf stomatal closure and other responses (Farooq et al., 2009). As shown in **Figure 4C** and **Figure 6F, G**, HcNFYA1 increased ABA content in transgenic *Arabidopsis* under salt and drought stress by activating the expression of *NCED3* and *ABA1*, the key genes for ABA synthesis (José et al., 2005; Ren et al., 2007). 35S:*HcNFYA1 Arabidopsis* increased sensitivity to ABA in comparison with the WT (**Figure 7**), because HcNFYA1 promoted the expression of *ABF1*, *ABI5*, and *RAB18* genes (**Figure 6H-J**), which are positively regulated by ABA signaling (Choi et al., 2000; Sonia et al., 2016). (ii) ROS homeostasis. ROS can be accumulated excessively under salt and drought stress, and plants have evolved antioxidant systems to scavenge reactive oxygen species and thus maintain ROS homeostasis (Apel and Hirt, 2004). For example, ZmmiR169q/NFYA8 conferred salt tolerance in maize by maintaining ROS homeostasis (Xing et al., 2021). 35S:*HcNFYA1 Arabidopsis* has higher antioxidant enzyme activities and more expressions of corresponding enzyme genes than those of WT and 35S:*HcmiR169b Arabidopsis* subjected to salt and drought stress (**Figure 5I-K, M-O**), resulting in less accumulation of reactive oxygen species than the WT (**Figure 5B, C, F, G**). This suggested that HcNFYA1 confers salt and drought tolerance by maintaining ROS homeostasis. (iii) Cell membrane stability. When plants are exposed to salt and drought stress, the water potential within the cell becomes out of balance, causing cell membrane rupture (Farooq et al., 2009). In salt stress, HcNFYA1 regulated Na^+^ transport by increasing the expression of genes such as *NHX1* and *SOS3* (**Figure 6C, D**), thus improving the stability of the membrane system (Shi et al., 2002; Barragán et al., 2012). HcNFYA1 also activated the expression of *LEA3* and *P5CS* to enhance the accumulation of soluble proteins and osmolytes in plants (**Figure 3H**, **Figure 5H, L**, **Figure 6B**), thus enhancing the osmotic adjustment ability and increasing the salt and drought resistance (Székely et al., 2008; Duan et al., 2012).

All together, our research indicates that under salt and drought stress, the expression of HcmiR169b and *HcNFYA1* in *Halostachys caspica* has a significantly negative relationship, HcmiR169b was repressed and *HcNFYA1* was increased. Transcription factor HcNFYA1 confers plant tolerance to salt and drought stresses through multiple pathways (**Figure 8**). These results enrich our understanding of the environmental adaptation mechanisms for the dominant plant *H. caspica* in extremely arid and saline environments and have crucial theoretical significance. However, application of the HcmiR169b/HcNFYA1 module to the molecular breeding of crops still requires a deeper understanding of their roles in plant growth and development.

## Data availability statement

The original contributions presented in the study are included in the article/**Supplementary Material**. Further inquiries can be directed to the corresponding author.

## Author contributions

JJ and YZ designed the experiments, analyzed the data, and wrote the manuscript. JJ, SZ, FC, XH, and QL performed the experiments. All authors contributed to the article and approved the submitted version.
