# Supplementary material for: The miR169b/NFYA1 module from the halophyte Halostachys caspica endows salt and drought tolerance in Arabidopsis through multi-pathways
Source: Front Plant Sci. 2023 Jan 10;13:1026421. doi: 10.3389/fpls.2022.1026421 (PMC9886095; doi:10.3389/fpls.2022.1026421)
Supplement: Supplementary file 1 [file DataSheet_1.docx]

**Supplementary Table S1 Primer sequences used in this study**

| **Primer name** | **Primer sequence (5’-3’)** | **Aim of amplifying genes with primers** |
| --- | --- | --- |
| RNA Adapter | ACUGAUGGCGAUGAAUGAACACUGCGUUUGCUGGCUUUGAUGAAA | RNA adapter in RLM-RACE |
| RACE-GSP | GCCAGCAAACGCAGT | Reverse transcription primers in RLM-RACE |
| RACE-HcNFYA1-F1 | CCAACGGCTCAGGAGGAAGTAGA | Primers for nested PCR amplification in RLM-RACE |
| RACE-Adapter-R1 | CATCAAAGCCAGCAAACGCAGTGT |  |
| RACE-HcNFYA1-F2 | GTCTAACAAGCAATGGGGTAGCGGCATGTC |  |
| RACE-Adapter-R2 | GCAAACGCAGTGTTCATTCATCGCCATCAG |  |
| RT-Stem-loop-HcmiR169b | GTCGTATCCAGTGCAGGGTCCGAGGTATTCGCACTGGATACGACTCGGCA | Stem-loop method of reverse transcription of HcmiR169b |
| qRT-HcmiR169b-F | GGCACGCCAAGGATGACT | qRT-PCR of HcmiR169b |
| qRT-HcmiR169b-R | GCAGGGTCCGAGGTATTC |  |
| qRT-HcU6-F | GGGGACATCCGATAAAATTGG | Reverse transcription and qRT-PCR of *HcU6* |
| qRT-HcU6-R | ACCATTTCTCGATTTGTGCGT |  |
| qRT-HcNFYA1-F | TTCACAGCATCCGCAACT | qRT-PCR of *HcNFYA1* |
| qRT-HcNFYA1-R | CCATAGGCAGCCATCATTC |  |
| qRT-HcUBQ10-F | CATATACTAAAGGAACAGTGGAGCCC | qRT-PCR of *HcUBQ10* |
| qRT-HcUBQ10-R | CACCATGGCATACTTCTGAGTCC |  |
| pCAMBIA1301-1-HcNFYA1-F (*Kpn*Ⅰ) | GGGGTACCTTGTCATTGAAGCATG | Vector construction for HcNFYA1 subcellular localization in onion cells |
| pCAMBIA1301-1-HcNFYA1-R (*Sal*Ⅰ) | GTCGACCACTCGATATGCTC |  |
| pGBKT7-HcNFYA1-F (*Eco*RI) | CGGGAATTCATGCCATCAAAGTCT | Vector construction for analysis of HcNFYA1 transcriptional activation activity in yeast |
| pGBKT7-HcNFYA1-R (*Bam*HI) | CGCGGATCCTCAGATGGCTAGA |  |
| pCAMBIA2300-HcmiR169b-F (*Kpn*Ⅰ) | GGTACCCGTAAGAGAGAGATACATG | Vector construction for heterologous expression of HcmiR169b in *Arabidopsis* |
| pCAMBIA2300-HcmiR169b-R (*Sal*Ⅰ) | GTCGACGGTAACTTAATTCATCTTAC |  |
| pCAMBIA2300-HcNFYA1-F (*Kpn*Ⅰ) | GGGGTACCTTGTCATTGAAGCATG | Vector construction for heterologous expression of *HcNFYA1* in *Arabidopsis* |
| pCAMBIA2300-HcNFYA1-R (*Bam*HⅠ) | CGGGATCCCACTCGATATGCTC |  |
| qRT-AtU6-F | CACAATACCATCCTACATGATTAAT | Reverse transcription and qRT-PCR of *AtU6* |
| qRT-AtU6-R | ATTAATCATGTAGGATGGTATTGTG |  |
| qRT-AtActin-F | TATGAATTACCCGATGGGCAAG | qRT-PCR of *AtActin* |
| qRT-AtActin-R | TGGAACAAGACTTCTGGGCAT |  |
| qRT-AtNFYA1-F | CAGAGCCCTGGTGGAAAAACAACT | qRT-PCR of *AtNFYA1* |
| qRT-AtNFYA1-R | TTTCTCTTCCCGGTTTTGATTGCAT |  |
| qRT-AtNFYA5-F | CATCTTGGGGAAACTCAATGCCTA | qRT-PCR of *AtNFYA5* |
| qRT-AtNFYA5-R | CTTCTTTCCTTTGAAACACTTGCAT |  |
| qRT-AtP5CS-F | GGGACAAGTTGTGGATGGAGAC | qRT-PCR of *AtP5CS* |
| qRT-AtP5CS-R | TGGTACAAACCTCAAGGAACAC |  |
| qRT-AtPOD-F | CCAAACTCTTCGTGGACTATGC | qRT-PCR of *AtPOD* |
| qRT-AtPOD-R | AACTCTTGGTCGCTCTGGAT |  |
| qRT-AtAPX-F | CTCTGGGACGATGCCACAAG | qRT-PCR of *AtAPX* |
| qRT-AtAPX-R | CTCGACCAAAGGACGGAAAA |  |
| qRT-AtCAT-F | GCAACTACCCCGAGTGGAAA | qRT-PCR of *AtCAT* |
| qRT-AtCAT-R | TGTTCAGAACCAAGCGACCA |  |
| qRT-AtRD29A-F | GCCGAGAAACTTCAGATTGG | qRT-PCR of *AtRD29A* |
| qRT-AtRD29A-R | CCATTCCTCCTCCTCCTTTC |  |
| qRT-AtLEA3-F | GATTGACCCGGCTGAGCTACGA | qRT-PCR of *AtLEA3* |
| qRT-AtLEA3-R | AGATGGGATTCACCACAAAAGA |  |
| qRT-AtSOS3-F | GACCGCAAAAACGACGGAAA | qRT-PCR of *AtSOS3* |
| qRT-AtSOS3-R | ACAGTACACAAGGCAAGTCCA |  |
| qRT-AtNHX1-F | AGTGTCGAAACTGCCTTCGT | qRT-PCR of *AtNHX1* |
| qRT-AtNHX1-R | CCAGTGCCTAGCCCAATCAA |  |
| qRT-AtDREB2A-F | GACCTAAATGGCGACGATGT | qRT-PCR of *AtDREB2A* |
| qRT-AtDREB2A-R | TCGAGCTGAAACGGAGGTAT |  |
| qRT-AtABF1-F | TCCGTGGTAAGAAGGTGAAGTC | qRT-PCR of *AtABF1* |
| qRT-AtABF1-R | TGTAAGCGTCTCTGTAATTTCTCC |  |
| qRT-AtRAB18-F | GAGCACCACGAGAAGAAGG | qRT-PCR of *AtRAB18* |
| qRT-AtRAB18-R | GCACAATACAACGACCGAATG |  |
| qRT-AtABI5-F | ATGATCAAGAACCGCGAGTCTGC | qRT-PCR of *AtABI5* |
| qRT-AtABI5-R | CGGTTGTGCCCTTGACTTCAAAC |  |
| qRT-AtNCED3-F | CAGCTTGTAGCTTTTGGGCTGTA | qRT-PCR of *AtNCED3* |
| qRT-AtNCED3-R | TAACAGAAACCAGCTGAGCTCGA |  |
| qRT-AtABA1-F | CGTGCGGTTGGAGAAGATGTGAT | qRT-PCR of *AtABA1* |
| qRT-AtABA1-R | TCTCAGAATGGCTTCCTCCTCAGT |  |

**Supplementary Table S2** Functions of NFYA family members in plant’s salt and drought stress

| **Species** | **Gene** | **Functions in plants** | **Reference** |
| --- | --- | --- | --- |
| *A. thaliana* | *AtNFYA5* | Negatively regulated by AtmiR169a, enhancing plant drought resistance. | Li et al., 2008 |
| *A. thaliana* | *AtNFYA1* | Activating ABA signaling during germination to sensitize plant to salt stress. | Li et al., 2013 |
| *G. max* | *GmNFYA3* | Negatively regulated by GmmiR169c, enhancing plant drought tolerance and diminishing plant salt tolerance. | Ni et al., 2013 |
| *G. max* | *GmNFYA5* | Enhancing drought tolerance in plant through ABA-dependent pathway. | Ma et al., 2020b |
| *G. max* | *GmNFYA13* | Enhancing drought and salt tolerance in plant through ABA-dependent pathway. | Ma et al., 2020a |
| *P. trichocarpa* | *PtNFYA6* | Inhibited by PtmiR169o to make plant sensitive to drought. | Jiao et al., 2021 |
| *P. trichocarpa* | *PtNFYA9* | Negative regulation of plant seed germination under abiotic stress and positive regulation of drought tolerance in adult plant. | Lian et al., 2018 |
| *Z. mays* | *ZmNFYA8* | Inhibited by ZmmiR169q, enhancing plant salt tolerance by maintaining ROS homeostasis. | Xing et al., 2021 |
| *O. sativa* | *OsNFYA7* | Enhancing drought tolerance in plant through ABA-independent pathway. | Lee et al., 2015 |


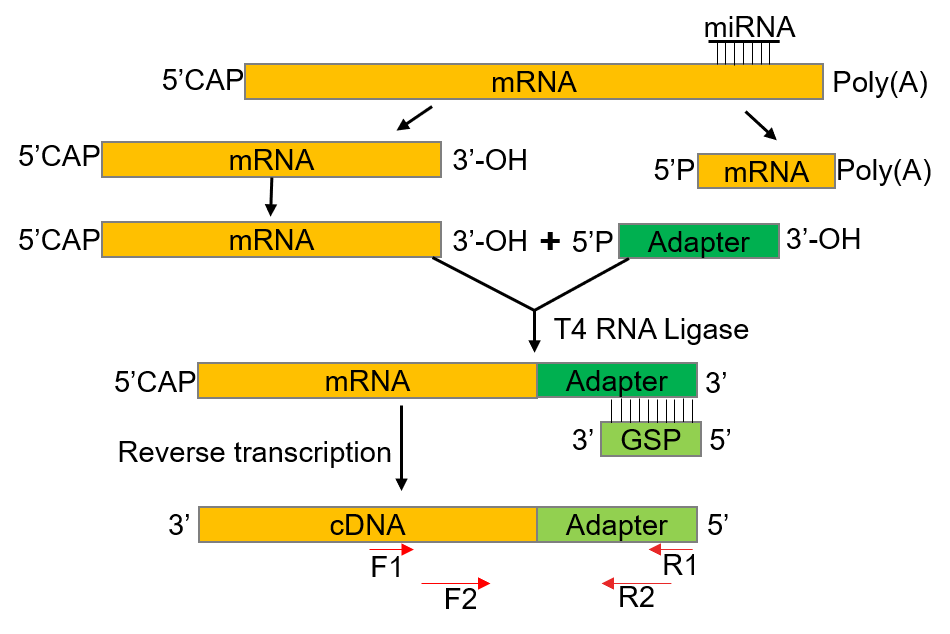
**Supplementary Figure S1 Flow chart for RLM-RACE analysis.**

**
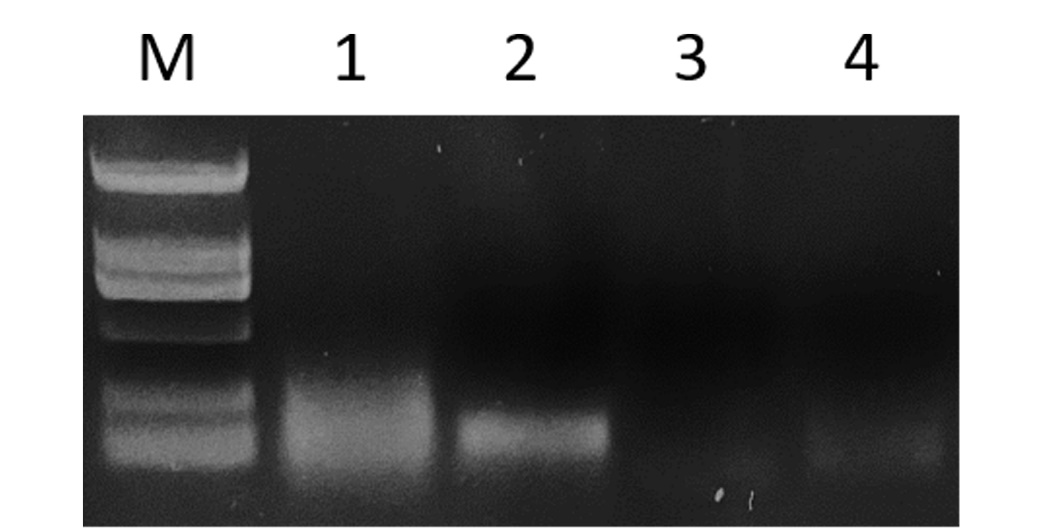
Supplementary Figure S2 Agarose gel electrophoresis assay**

**for RLM-RACE experiment.**

M: DNA Marker DL2000. 1: First round PCR products. 2: Second round nested PCR products. 3-4: Negative controls.

**
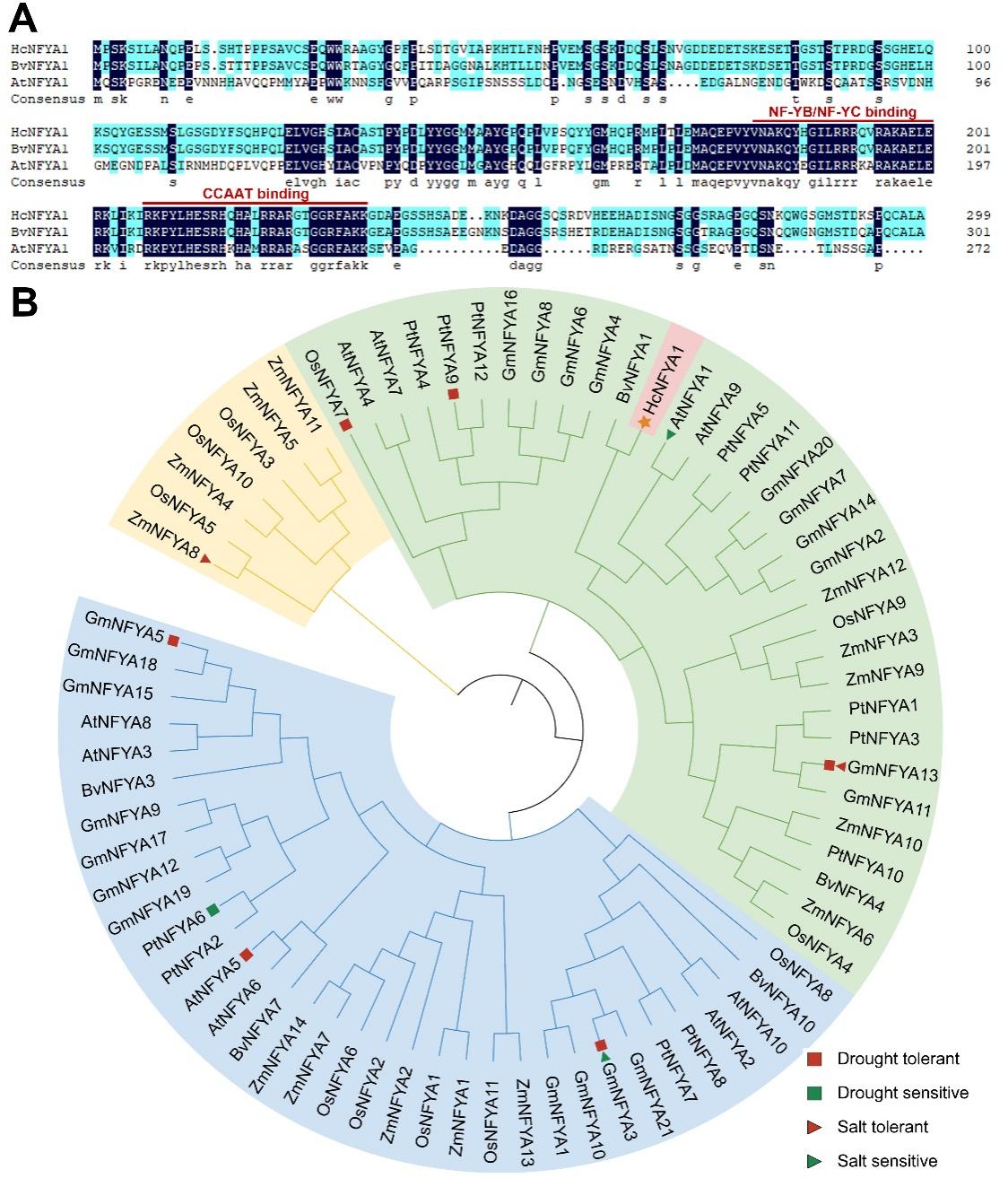
Supplementary Figure S3 HcNFYA1 bioinformatic analysis.**

**(A)** HcNFYA1 amino acid sequence analysis. **(B)** HcNFYA1 phylogenetic analysis.


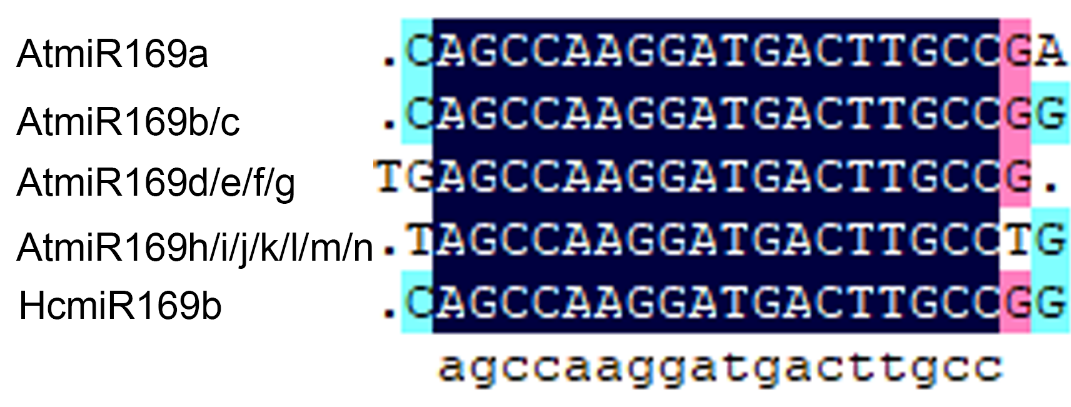
**Supplementary Figure S4 Comparison of HcmiR169b with AtmiR169 family mature sequences.**

**
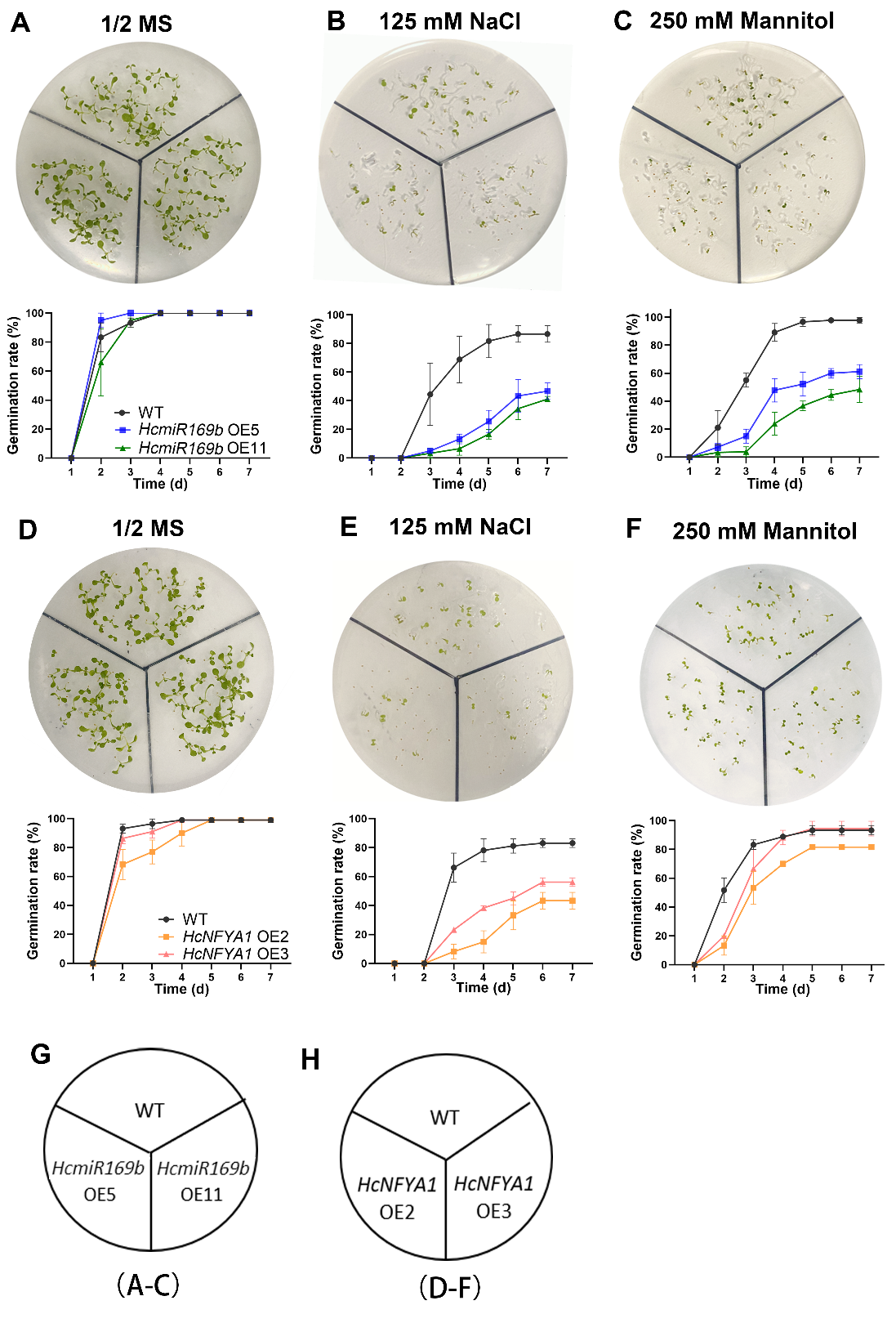
Supplementary Figure S5 *Halostachys caspica* miR169b/NFYA1 module affects the seed germination of *Arabidopsis* under salt and drought stresses.**

**(A-C)** Germination phenotypes and germination curves of the WT and 35S:*HcmiR169b* *Arabidopsis* cultured in 1/2 MS **(A)**, 125 mM NaCl **(B),** and 250 mM Mannitol **(C)** medium for 7 d. **(D-F)** Germination phenotypes and germination curves of the WT and 35S:*HcNFYA1 Arabidopsis* cultured in 1/2 MS **(D)**, 125 mM NaCl **(E)**, and 250 mM Mannitol **(F)** medium for 7 d. **(G-H)** Layout designs of figure A-C **(G)** and D-F **(H)**. Data were shown as the means ± SD of three independent experiments.
